# The effect of extended early rehabilitation on the treatment outcome of patients with moderate and severe traumatic brain injury

**DOI:** 10.3389/fnhum.2025.1637199

**Published:** 2025-09-08

**Authors:** Nataša Keleman, Dragana Dragičević-Cvjetković, Aleksandra Mikov, Dragomir Radošević, Ðula Ðilvesi, Vladimir Mrđa, Rastislava Krasnik

**Affiliations:** ^1^Clinical Rehabilitation Service, University Clinical Center of the Republic of Srpska, Banja Luka, Bosnia and Herzegovina; ^2^Institute of Physical Medicine, Rehabilitation and Orthopedic Surgery “Dr Miroslav Zotović”, Banja Luka, Bosnia and Herzegovina; ^3^Faculty of Medicine, University of Banja Luka, Banja Luka, Bosnia and Herzegovina; ^4^Faculty of Medicine, University of Novi Sad, Novi Sad, Serbia; ^5^Institute for Child and Youth Health Care of Vojvodina, Novi Sad, Serbia; ^6^Klinikum Bad Gastein, Bad Gastein, Austria; ^7^Clinic of Neurosurgery, University Clinical Center of Vojvodina, Novi Sad, Serbia; ^8^Clinic for Anesthesiology and Intensive Care, University Clinical Center of the Republic of Srpska, Banja Luka, Bosnia and Herzegovina

**Keywords:** traumatic brain injury, epidemiology, early rehabilitation, clinical outcome, Intensive Care Unit

## Abstract

**Introduction:**

Traumatic brain injury (TBI) is one of the leading causes of morbidity, disability and mortality in all age groups. The functional disability left by TBI, is significant for the patient, family and society. Treatment and early rehabilitation are crucial to the overall level of recovery.

**Objective:**

To compare the impact of extended early rehabilitation of patients with moderate and severe traumatic brain injury on the outcome of treatment.

**Material:**

A prospective experimental study comprised 124 patients with moderate and severe TBI, randomly divided into two groups. The patients of the experimental group had kinesitherapy seven days a week, twice a day for 45 min, and the patients of the control group had kinesitherapy seven days a week, twice a day for 30 min. The follow-up parameters were: age, sex, mechanism of injury, associated injuries, comorbidities, Glasgow Coma Scale (GCS) on admission and discharge, early rehabilitation interruptions, presence of tracheostomy, decubitus ulcers, nasogastric tube, diapers and urinary catheter at the discharge from the Intensive Care Unit and discharge from the hospital, and mortality.

**Results:**

124 patients were randomly divided into two groups of 62 patients each. The male gender dominated 95 (76.6%). The average age was Me = 62.00 (IQR = 36.0) years. There was no statistically significant difference between the groups in gender, age, mechanism of injury, associated injuries and comorbidities. GCS on admission; 68 (54.8%) patients had severe, 56 (46.2%) moderate TBI, at discharge: 22 (17.7%) had severe, 11 (8.9%) moderate and 91 (73.4%) mild TBI. At discharge from the ICU, the presence of tracheostomy was (13 vs. 19), (*p* = 0.218), nasogastric tube (33 vs. 45), (*p* = 0.026) and decubitus ulcers (0 vs. 4), (*p* = 0.042) were less in the experimental group. At discharge from the hospital, tracheostomy (6 vs. 16), (*p* = 0.019), urinary catheter (38 vs. 48), (*p* = 0.051), diapers (29 vs. 40), (*p* = 0.047) were less in the experimental group. There were 5 deaths in both groups. There was no statistically significant difference in early rehabilitation interruptions between groups (5 vs. 6).

**Conclusion:**

Extended early TBI rehabilitation is safe, effective and contributes to a positive outcome of treatment.

## 1 Introduction

There is still no universally accepted definition of traumatic brain injury (TBI). According to the World Health Organization, TBI is an acute brain injury, which occurs as a result of an external mechanical force on the head or a penetrating craniocerebral injury, excluding the effects of drugs, alcohol and medications (Carroll et al., 2004).

Traumatic brain injury is one of the leading causes of morbidity, disability and mortality in all age groups. Worldwide, more than 50 million people experience TBI every year. TBI is not only the leading cause of death in the population of under 45 years, but also the leading cause of death in trauma in Europe (Tagliaferri et al., 2006). Data on the incidence of TBI in Europe are very wide ranging. Reported incidence and mortality rates of TBI vary among countries and regions and range from 47.3 to 849 per 100,000 inhabitants per year. Mortality rates are from 9 to 28.10 per 100,000 inhabitants per year (Peeters et al., 2015; Brazinova et al., 2021). A total of 57 studies recorded the seven most common mechanisms of injury of TBI: traffic trauma, falls, violence, sports injuries, accidents at home or at work, and suicide or attempted suicide (Brazinova et al., 2021).

The Glasgow Coma Scale (GCS), which is used to assess the level of consciousness immediately after a brain injury, is generally accepted for assessing the level of consciousness after TBI. Considering the total GCS, all brain injuries are divided into mild (GCS ≥ 13), moderate (GCS 9–12) and severe (GCS ≤ 8) (Teasdale and Jennett, 1974; Giacino et al., 2014; Mikolić et al., 2021a).

TBI leads to various neurological symptoms such as impaired consciousness and motor control, sensory dysfunction, cognitive disorders and breathing disorders. These changes are accentuated by the changes that occur due to immobility, mechanical ventilation (MV), sedation and relaxation during hospitalization (Mayo Clinic, 2023). The central nervous system has the ability to recover and adapt to secondary compensatory mechanisms.

For a long time, there was a generally accepted opinion in medical circles that a patient with TBI, especially if the patient is on MV, is “too weak and sick” for early rehabilitation (Bailey et al., 2007). That is why the implementation of early rehabilitation in everyday clinical practice was a big challenge (Dubb et al., 2016). Thanks to the progress of medicine, this opinion has changed significantly, because an increasing number of patients survive TBI, but unfortunately with a high rate of disability. Neurological damage and functional impairment can persist for years, even for life. Therefore, there has been an increased focus of interest on the effectiveness of early rehabilitation in improving physical, cognitive, psychosocial, and functional impairments after TBI. Rehabilitation after TBI should be started as early as possible. The aim of early rehabilitation is to improve motor and functional recovery, while preventing secondary complications (pneumonia, atelectasis, muscle atrophy, decubitus ulcer, deep vein thrombosis, contractures and others). It is recommended that the intensity and duration of early rehabilitation must be individually dosed depending on the patient’s general condition and capabilities with a multidisciplinary approach. Although numerous strategies have been developed to optimize early rehabilitation (ER), there are still no clearly defined global guidelines on the intensity, frequency and duration of ER in TBI (Gerber et al., 2021; Rai et al., 2021). The safety of early rehabilitation has been a stumbling block in the introduction of early rehabilitation after TBI. In a meta-analysis by Nydahl et al. (2017) that included 48 publications, with 7,546 patients (23,351 sessions), there were 583 adverse events (2.6%).

The benefits of early rehabilitation after TBI have been proven by numerous studies (Qannam et al., 2017; Tipping et al., 2017; Lang et al., 2020). Early rehabilitation provides significantly better functional outcome than delayed rehabilitation. Early rehabilitation is associated with the improved outcome, in terms of reduced coma and hospitalization duration, better cognitive level at discharge, better score on functional scales and greater chances of returning home. There is a positive correlation between the intensity of early rehabilitation and the outcome, and it has been proven that it has a positive effect on neuroplasticity (Andelic et al., 2012; Tipping et al., 2017; Fan et al., 2020; Rai et al., 2021).

Objective of the study is to compare the impact of extended early rehabilitation of patients with moderate and severe traumatic brain injury on the outcome of treatment.

## 2 Material and methods

After the approval of the Ethics Committee of the University Clinical Center, Banja Luka, the Republic of Srpska, the research was conducted as a prospective experimental study that comprised 124 patients of both sexes, aged over 18 years with moderate and severe traumatic brain injury, who were admitted to the Clinic for Anesthesia and Intensive Care and the Clinic for Neurosurgery of the University Clinical Center of the Republika Srpska Banja Luka, in the period from 05.09.2022 to 15.12.2023. The patients were divided into two groups, 62 in each group, randomized (every second patient), that were included in early rehabilitation after being examined by a specialist in physical medicine and rehabilitation.

The first group (experimental) consisted of patients who were involved in early rehabilitation seven days a week, twice a day, lasting 45 min per session, i.e., 90 min a day. The second group (control) consisted of patients who were involved in standard early rehabilitation, 7 days a week, twice a day, lasting 30 min per session, i.e., 60 min a day. All patients had an identical kinesitherapy implementation plan, which included positioning, an anti-decubitus program, passive and actively assisted exercises to maintain and increase the range of motion in all segments of the upper and lower extremities, as well as respiratory kinesitherapy.

The evaluation of the patients included the registration of the following data: sociodemographic data: gender, age, mechanism of brain injury (traffic trauma, falls, other), clinical characteristics–associated injuries (locomotor system, chest, abdomen, cervical spine and polytrauma), comorbidities (hypertension, diabetes mellitus, asthma), GCS on admission and discharge, data on the termination of early rehabilitation. The presence of the following parameters at the time of discharge from the Intensive Care Unit and discharge from the hospital was analyzed: tracheostomy, urinary catheter, nasogastric tube, diapers, decubitus ulcers and mortality.

The criteria for the inclusion of patients in the study were: persons of both sexes with TBI verified by computed tomography (CT), aged 18 years or older and hemodynamically stable for early rehabilitation [heart rate 60–130/minute, mean arterial pressure 60–100 mm Hg, respiratory rate 5–40 per minute, peripheral oxygen saturation of > 88%, if the patient is on mechanical ventilation the fraction of inhaled oxygen of FiO_2_ < of 60% is required and a positive end-expiratory pressure (PEEP) of < 10 cm H_2_O, an artificial airway is properly secured]. During early rehabilitation, an increase in heart rate by 20 beats per minute and an increase in mean arterial pressure by 20 mm Hg is allowed (Sommers et al., 2015; Jang et al., 2019).

The criteria for excluding patients from the study were: persons with TBI who were hemodynamically unstable (heart rate < 50 or > 130 per minute, systolic blood pressure < 90 or > 180 mm Hg, acute myocardial infarction or occurrence of arrhythmias), tachypnea or bradypnea, oxygen saturation of < 88%, uncertain air flow, acute bleeding, unstable bone fractures, deep vein thrombosis and pulmonary embolism, body temperature of > 38 °Ñ.

Criteria for termination of early rehabilitation–signs of intolerance: sweating, tachypnea > 40 per minute, drop in oxygen saturation below 88%, mean arterial pressure < 50 or > 100 mm Hg, pulse < 60 or > 130 per minute, and deterioration of neurological status (deterioration of GCS by two points, development of mydriasis and development of focal neurological deficit) (Sommers et al., 2015; Jang et al., 2019).

The total sample size was determined at *N* = 124, that is of 62 respondents in each group. The sample calculation was made for a dichotomous outcome, using the χ^2^ test, for two independent samples, with an alpha error value of 0.05 (α = 0.05) and a beta error value of 0.2 (consequently, the power of the study is 80%), for the number of respondents in 1:1 groups, using G*Power 3.1.2. computer program (Faul et al., 2007). The sample size of 124 participants (62 per group) was determined *a priori* based on the assumption of detecting a moderate effect (Cohen’s *d* = 0.5) for a dichotomous primary outcome. Although multiple outcomes were examined, the study primarily aimed to evaluate functional and clinical recovery parameters at ICU and hospital discharge, including Glasgow Coma Scale and the presence of tracheostomy, nasogastric tube, or decubitus ulcers. Due to the presence of multiple secondary outcomes, we also report effect sizes for key continuous and categorical variables to supplement *p*-values.

## 3 Statistical data processing

The results obtained by the research were statistically processed with an adequate selection of statistical methods, in order to provide an optimal model of understanding the influence, dependence and differences between the analyzed data obtained in the research.

Descriptive statistics were used to summarize the sample characteristics, including frequencies and percentages for categorical variables, as well as minimum and maximum values, medians (Me), and interquartile ranges (IQR) for continuous variables. Given that numerical variables significantly deviated from the normal distribution, as determined by distributional assessments, only non-parametric tests were applied in the analysis. The Mann–Whitney U test was used to compare differences between the experimental and control groups, while the Wilcoxon Signed Ranks Test was employed to assess differences between two time points within the same group (e.g., GCS on admission vs. discharge).

To examine group differences in categorical variables, the Chi-square test was primarily used. However, in cases where more than 20% of the cells in the contingency tables had expected frequencies less than 5, the likelihood ratio test was applied instead. To control for the risk of false positives due to multiple comparisons, the *p*-values were adjusted using the False Discovery Rate (FDR) correction according to the Benjamini–Hochberg procedure.

Finally, logistic regression analyses were conducted to estimate the probability of clinical outcomes according to group assignment (experimental vs. control). Both unadjusted and adjusted models were tested, with adjustment for potential confounding variables such as age (continuous), comorbidities (hypertension, diabetes, bronchial asthma), injury severity (locomotor, cervical spine, chest, abdomen, polytrauma), and mechanism of TBI occurrence. All binary variables were coded as 1 = yes and 0 = no, with the control group set as the reference category.

Statistical analysis was carried out using the IBM SPSS Statistics for Windows, ver. 24.0 (IBM Corp., Armonk, NY, USA).

## 4 Results

The research included 95 (76.6%) men and 29 (23.4%) women. The distribution of patients by age category shows that 18 (14.5%) patients are between 18 and 30 years old, 14 (11.3%) between 31 and 40 years old, 11 (8.9%) between 41 and 50 years old, 17 (13.7%) between 51 and 60 years old, 24 (19.4%) between 61 and 70 years old, 17 (13.7%) between 71 and 80 years old, while 23 (18.5%) patients are between 81 and 90 years old. The average age of all patients were Me = 62.00 years (IQR = 36.0). The youngest patient is 18, and the oldest is 90 years old. There were no statistically significant differences between the experimental and control groups regarding gender (*p* = 0.832) and age structure (*p* = 0.211). The average age of patients in the experimental group were Me = 64.0 years (IQR = 42.7), while in the control group it was Me = 58.5 years (IQR = 30.2), *p* = 0.506 (Table 1).

**TABLE 1:** Sex and age characteristics in the experimental and control groups.

	Experimental	Control	*p*-value	FDR corrected *p*-value	All
	(*n* = 62)	(*n* = 62)			(*n* = 124)
**Sex, *n* (%)**			0.832^a^	0.876	
Male	48 (77.4%)	47 (75.8%)			95 (76.6%)
Female	14 (22.6%)	15 (24.2%)			29 (23.4%)
**Age (years), Min–Max, Me (IQR)**	18.00–90.00, 64.0 (42.7)	21.00–88.00, 58.5 (30.2)	0.506^b^		18.00–90.00, 62.0 (36.0)
**Age categories, *n* (%)**			0.211^c^	0.545	
18–30	12 (19.4%)	6 (9.7%)			18 (14.5%)
31–40	6 (9.7%)	8 (12.9%)			14 (11.3%)
41–50	3 (4.8%)	8 (12.9%)			11 (8.9%)
51–60	6 (9.7%)	11 (17.7%)			17 (13.7%)
61–70	11 (17.7%)	13 (21.0%)			24 (19.4%)
71–80	9 (14.5%)	8 (12.9%)			17 (13.7%)
81–90	15 (24.2%)	8 (12.9%)			23 (18.5%)

^a^Chi-square test, ^b^Mann–Whitney U test, ^c^likelihood ratio test. *n*, number of respondents; %, percentage; Min, minimum; Max, maximum; Me, median; IQR, interquartile range.

The most frequent mechanism of TBI were falls, in 64 (51.6%) patients. Traffic traumas were registered in 37 (29.8%) patients. Other categories of injury mechanisms included 23 (18.5%) patients. In the analysis of the frequency of TBI mechanisms in the experimental and control groups, the following results were recorded: falls are the most common mechanism of injury, present in 38 (61.3%) patients from the experimental group and 26 (41.9%) from the control group. Traffic trauma was recorded in 15 (24.2%) patients from the experimental group and 22 (35.5%) from the control group. This difference was not statistically significant (*p* = 0.097), which indicated a similar distribution of mechanisms between the groups (Table 2).

**TABLE 2:** Frequency of mechanisms of traumatic brain injury occurrence in the experimental and control group.

	Experimental	Control	*p*-value	FDR corrected *p*-value	All
	(*n* = 62)	(*n* = 62)			(*n* = 124)
**Mechanism of TBI occurence, *n* (%)**			0.097^a^	0.323	
Traffic traumatism	15 (24.2%)	22 (35.5%)			37 (29.8%)
Falls	38 (61.3%)	26 (41.9%)			64 (51.6%)
Other	9 (14.5%)	14 (22.6%)			23 (18.5%)

^a^Chi-square test. *n*, number of respondents; %, percentage; TBI, traumatic brain injury.

In both groups of patients, a similar number of patients have locomotor system injuries, 14 (22.6%) in the experimental and 12 (19.4%) in the control group, cervical spine injuries 5 (8.1%) in the experimental and 7 (11.3%) in the control group, chest injuries 15 (24.2%) in the experimental and 17 (27.4%) in the control group, abdomen injuries 5 (8.1%) in the experimental and 2 (3.2%) in the control group. Polytrauma was registered in 21 (33.9%) patients in the experimental group and 25 (40.3%) in the control group. The differences were not statistically significant even before and after FDR correction (all *p*-values > 0.05) (Table 3).

**TABLE 3:** Associated injuries in the experimental and control group.

	Experimental	Control	*p*-value	FDR corrected *p*-value	All
	(*n* = 62)	(*n* = 62)			(*n* = 124)
**Associated injuries, *n* (%)**					
Locomotor system	14 (22.6%)	12 (19.4%)	0.659^a^	0.851	26 (21.0%)
Cervical spine	5 (8.1%)	7 (11.3%)	0.543^b^	0.835	12 (9.7%)
Chest	15 (24.2%)	17 (27.4%)	0.681^a^	0.851	32 (25.8%)
Abdomen	5 (8.1%)	2 (3.2%)	0.439^b^	0.762	7 (5.6%)
Polytrauma	21 (33.9%)	25 (40.3%)	0.457^a^	0.762	46 (37.1%)

^a^Chi-square test, ^b^likelihood ratio test. *n*, number of respondents; %, percentage.

In both groups of patients, a similar percentage of patients had hypertension as a comorbidity, in the experimental 37 (59.7%) and 41 (66.1%) in the control group, diabetes 12 (19.4%) in the experimental and 13 (21.0%) in the control group, and bronchial asthma 12 (19.4%) in the experimental and 14 (22.6%) in the control group. There were no statistically significant differences between the groups in any of the listed categories (all *p*-values > 0.05) (Table 4).

**TABLE 4:** Comorbidities, difference between the experimental and control group.

	Experimental	Control	*p*-value	FDR corrected *p*-value	All
	(*n* = 62)	(*n* = 62)			(*n* = 124)
**Comorbidities, *n* (%)**					
Hypertension	37 (59.7%)	41 (66.1%)	0.457^a^	0.762	78 (62.9%)
Diabetes	12 (19.4%)	13 (21.0%)	0.823^a^	0.876	25 (20.2%)
Bronchial asthma	12 (19.4%)	14 (22.6%)	0.659^a^	0.851	26 (21.0%)

^a^Chi-square test. *n*, number of respondents; %, percentage.

In the analysis of the differences between the experimental and control group on the parameters at discharge from the Intensive Care Unit, the results show the following: when it comes to the presence of a tracheostomy, 13 (21.0%) patients from the experimental and 19 (30.6%) from the control group had a tracheostomy. The difference were not statistically significant (*p* = 0.218). Regarding the nasogastric tube, 33 (53.2%) patients from the experimental group and 45 (72.6%) from the control group had a nasogastric tube. The difference between the groups was statistically significant (*p* = 0.026), but after FDR correction this difference is lost (*p* = 0.173). All patients in both groups had diapers and a urinary catheter. No decubitus ulcers were registered in the experimental group, while 4 patients (6.5%) were recorded in the control group. The difference is statistically significant (*p* = 0.017) before, but not after FDR correction (*p* = 0.170). Differences in parameters between the experimental and control group at hospital discharge were tested. In the experimental group, 6 (9.7%) patients had a tracheostomy, while in the control group, that number was 16 (25.8%). The difference between groups was statistically significant (*p* = 0.017), but not after FDR correction (*p* = 0.170). When it comes to decubitus ulcers, the differences were not statistically significant (*p* = 0.729), that was, 4 (6.5%) patients from the experimental group and 5 (8.1%) from the control group had pressure ulcers. The presence of a urinary catheter was more common in the control group 48 (77.4%) compared to 38 (61.3%) in the experimental group. The difference was at the limit of statistical significance (*p* = 0.051), but not after FDR correction (*p* = 0.204). When it comes to the presence of diapers, it was also more common in the control group 40 (64.5%) compared to 29 (46.8%) in the experimental group, and the difference is statistically significant (*p* = 0.047), while it lost statistical significance after FDR correction (*p* = 0.204). Regarding the nasogastric tube, no statistically significant difference was noted between the groups (*p* = 0.363). Therefore, considering that after the FDR correction, all statistical significance was lost, the differences should be taken with a grain of salt (Table 5).

**TABLE 5:** Parameters at discharge, difference between the experimental and control group.

	Experimental	Control	*p*-value	FDR corrected *p*-value	All
	(*n* = 62)	(*n* = 62)			(*n* = 124)
**Parameters at discharge from the Intensive Care Unit, *n* (%)**					
Tracheostomy	13 (21.0%)	19 (30.6%)	0.218^a^	0.545	32 (25.8%)
Urinary catheter	62 (100.0%)	62 (100.0%)	NA		124 (100.0%)
Nasogastric tube	33 (53.2%)	45 (72.6%)	***0.026*** ^a^	0.173	78 (62.9%)
Diapers	62 (100.0%)	62 (100.0%)	NA		124 (100.0%)
Decubitus ulcers	0 (0.0%)	4 (6.5%)	***0.017*** ^b^	0.170	4 (3.2%)
**Parameters at discharge from hospital, *n* (%)**					
Tracheostomy	6 (9.7%)	16 (25.8%)	***0.017*** ^b^	0.170	22 (17.7%)
Decubitus ulcers	4 (6.5%)	5 (8.1%)	0.729^b^	0.858	9 (7.3%)
Presence of urinary catheter	38 (61.3%)	48 (77.4%)	***0.051*** ^a^	0.204	86 (69.4%)
Presence of diapers	29 (46.8%)	40 (64.5%)	***0.047*** ^a^	0.204	69 (55.6%)
Nasogastric tube	10 (16.1%)	14 (22.6%)	0.363^a^	0.762	24 (19.4%)

^a^Chi-square test, ^b^likelihood ratio test. *n*, number of respondents; %, percentage; NA, not applicable. Statistically important result is marked bold.

Within the presentation of Glasgow Coma Scale (GCS) results at admission and discharge, no statistically significant difference was found between the experimental and control groups in any of the investigated metrics. Although the median GCS at admission was higher in the experimental group (Me = 9.0, IQR = 7.0) compared to the control group (Me = 3.0, IQR = 7.0), the difference was not statistically significant (*p* = 0.070). Also, the median GCS at discharge is Me = 15.0 (IQR = 1.25) in the experimental and Me = 14.0 (IQR = 4.25) in the control group, without a significant difference (*p* = 0.130). Categorical distribution of injury severity based on GCS also shows no significant differences between groups, neither at admission (*p* = 0.149) nor at discharge (*p* = 0.526). The largest number of patients in both groups at discharge was classified in the mild injury category (77.4% in the experimental and 69.4% in the control group). However, a statistically significant difference was recorded in the analysis of differences between admission and discharge both in the experimental (*p* < 0.001) and in the control group (*p* < 0.001) (Table 6).

**TABLE 6:** Glasgow Coma Scale on admission and at discharge, difference between the experimental and control group.

	Experimental	Control	*p*-value	FDR corrected *p*-value	All
	(*n* = 62)	(*n* = 62)			(*n* = 124)
GCS on admission, Min–Max, Me (IQR)	3.0–11.0, 9.0 (7.00)	3.0–11.0, 3.0 (7.00)	0.070^c^		3.0–11.0, 7.5 (7.00)
GCS at discharge, Min–Max, Me (IQR)	3.0–15.0, 15.0 (1.25)	3.0–15.0, 14.0 (4.25)	0.130^c^		3.0–15.0, 14.5 (3.75)
**GCS categories on admission, *n* (%)**			0.149^a^	0.454	
Severe injury	30 (48.4%)	38 (61.3%)			
Moderate injury	32 (51.6%)	24 (38.7%)			
Mild injury	0 (0.0%)	0 (0.0%)			
**GCS categories at discharge, *n* (%)**			0.526^b^	0.825	
Severe injury	10 (16.1%)	12 (19.4%)			22 (17.7%)
Moderate injury	4 (6.5%)	7 (11.3%)			11 (8.9%)
Mild injury	48 (77.4%)	43 (69.4%)			91 (73.4%)

^a^Chi-square test, ^b^likelihood ratio test, ^c^Mann–Whitney U test. *n*, number of respondents; %, percentage; NA, not applicable; GCS, Glasgow Coma Scale. The difference between GCS on admission and at discharge was statistically significant in both the experimental and control groups (*p* < 0.001 [Wilcoxon Signed Ranks Test]).

In the majority of the patients from both groups (experimental and control), early rehabilitation was not interrupted: 57 (91.9%) patients from the experimental group and 56 (90.3%) from the control group. When the interruptions of rehabilitation for various reasons is in matter, there are fewer cases and no statistically significant difference between the groups was found. As a reason for the termination of rehabilitation, worsening of the general condition was noted in 1 (1.6%) of the patient in each group; fever in 3 (4.8%) in each group; and hemodynamic instability in 1 (1.6%) in the experimental and 2 (3.2%) in the control group. In the majority of patient, early rehabilitation was carried out without interruption, and the reasons for interruption did not differ significantly between the groups (Table 7).

**TABLE 7:** Reason of interruption of early rehabilitation, difference between the experimental and control group.

	Experimental	Control	*p*-value	FDR corrected *p*-value	All
	(*n* = 62)	(*n* = 62)			(*n* = 124)
**Reason of interruption of early rehabilitation, *n* (%)**			0.952^a^	0.952	
There was no interruption	57 (91.9%)	56 (90.3%)			113 (91.1%)
Deterioration of the general condition	1 (1.6%)	1 (1.6%)			2 (1.6%)
Febrility	3 (4.8%)	3 (4.8%)			6 (4.8%)
Hemodynamic instability	1 (1.6%)	2 (3.2%)			3 (2.4%)

^a^Likelihood ratio test. *n*, number of respondents; %, percentage.

Table 8 shows the results of binary logistic regression, both unadjusted and adjusted for relevant confounding variables, to examine the association between group membership (experimental vs. control) and a range of clinical outcomes. In the unadjusted model, three effects on the dependent variable were identified. Patients in the experimental group were significantly less likely to have a nasogastric tube at ICU discharge (OR = 0.43; 95% CI: 0.20–0.91; *p* = 0.027) and less likely to have a tracheostomy at hospital discharge (OR = 0.31; 95% CI: 0.11–0.85; *p* = 0.023). Also, the presence of diapers at hospital discharge was significantly less frequent in the experimental group (OR = 0.48; 95% CI: 0.23–0.99; *p* = 0.048). However, after adjusting the model for confounding variables (age, comorbidities, injury severity and mechanism of TBI occurrence), none of the differences between the groups remained statistically significant. Although the direction of the effect remained similar in the unadjusted models, the confidence intervals widened and the *p*-values increased above the significance threshold. This change suggests that the differences observed in the unadjusted analyzes were likely partly the result of differences in the patients baseline characteristics. Although the unadjusted regression models indicated a potentially beneficial effect of prolonged rehabilitation on certain clinical outcomes, the results of the adjusted models emphasize the need for caution when interpreting differences between groups (Table 8).

**TABLE 8:** Unadjusted and adjusted odds ratios for clinical outcomes according to group assignment.

	Unadjusted model		Adjusted model	
Dependent variable	OR (95% CI)	*p*-value	OR (95% CI)	*p*-value
Tracheostomy at ICU discharge	0.60 (0.27–1.36)	0.220	0.85 (0.28–2.60)	0.772
Nasogastric tube at ICU discharge	0.43 (0.20–0.91)	***0.027***	0.47 (0.17–1.28)	0.137
Decubitus ulcers at ICU discharge	0.00 (0.00–NA)	0.997	0.00 (0.00–0.00)	0.995
Tracheostomy at hospital discharge	0.31 (0.11–0.85)	***0.023***	0.51 (0.13–2.09)	0.352
Decubitus ulcers at hospital discharge	0.79 (0.20–3.08)	0.730	1.29 (0.19–8.61)	0.795
Urinary catheter at hospital discharge	0.46 (0.21–1.01)	0.054	0.52 (0.19–1.40)	0.197
Presence of diapers at hospital discharge	0.48 (0.23–0.99)	***0.048***	0.53 (0.21–1.35)	0.183
Nasogastric tube at hospital discharge	0.66 (0.27–1.62)	0.365	0.49 (0.14–1.67)	0.252
GCS at discharge	1.46 (0.33–6.46)	0.619	1.35 (0.08–22.53)	0.836

All models were adjusted for the following variables: age (continuous), comorbidities (hypertension, diabetes, bronchial asthma), injury severity (locomotor, cervical spine, chest, abdomen, polytrauma), and mechanism of TBI occurrence (reference = other). All binary categorical variables were coded as 1 = yes and 0 = no (reference). Independent variable: group assignment (experimental/control [reference category]). Statistically important result is marked bold.

## 5 Discussion

Traumatic brain injuries, due to their consequences on the level of functional disability of patients and their quality of life, have great worldwide socio-medical and economic importance. There are two published epidemiological studies from Bosnia and Herzegovina (BiH) reporting 141 patients with severe traumatic brain injury in a two-year period (Dizdarević et al., 2006). Our sample consisted of 124 patients with moderate and severe TBI over a one-year period. In the majority of studies published so far, the male gender dominated, 55.4% vs. 67.6% vs. 74% (Heydari et al., 2019; Gao et al., 2020; Maiden et al., 2020). In the Republic of Srpska in 2022, 70% of all types of TBI were male patients (Keleman et al., 2023). In recent years, the proportion of females in TBI has been increasing. This can be explained by the increasing participation of women in traffic, sports, military service, as well as the increase in domestic violence, which is generally not reported (Monahan et al., 2017; Biegon, 2021). In a multicenter study that included 2,862 patients with TBI, 36% of women had mild and 26% had moderate or severe TBI (Mikolić et al., 2021b). According to the results of a study from Finland, 51.1% of respondents were female with TBI (Posti et al., 2020).

Our patients with TBI were on average 62.00 (IQR = 36.0) years old. The distribution of TBI is difficult to be compared between populations, because many studies examine single cohorts (children and/or adults of different age groups) (Brazinova et al., 2021). In studies that included patients of over 18 years of age, the average age was 58.8 years in Singapore, 54.5 years in Germany, 48 years in China, and 59.5 years in Italy (Lui et al., 2020; Schwenkreis et al., 2021; Yang et al., 2022; Ong et al., 2023). Studies that examined the elderly population of patients with TBI showed that the average age in Finland was 80.7 years, in China 72 years, and in Iran 70.61 years (Heydari et al., 2019; Posti et al., 2020; Finotti et al., 2021).

A total of 57 studies recorded the seven most common mechanisms of injury of TBI: traffic trauma, falls, violence, sports injuries, accidents at home or at work, and suicide or attempted suicide (Brazinova et al., 2021). In our study, falls dominate as the mechanism of TBI occurrence in 51.6% of patients, while traffic trauma was recorded in 29.8% of patients. The picture at the global level confirms these two mechanisms as leading ones with different ratios in various countries. However, in the past twenty years, there has been a change in the epidemiological characteristics depending on the age of the population and the level of development of the countries. A retrospective study in China of 4,229 patients recorded a fall as the most common mechanism of TBI in 42.1% of respondents (Zou et al., 2023). In Germany, among the population older than 50 years, in 71.3% of respondents TBI was caused by a fall, and among the population younger than 50 years, traffic trauma in 29%, and falls in 27% (Yang et al., 2022). In Singapore, a fall was recorded as the dominant cause of TBI in patients over 60 years of age in 74.5% of cases (Lui et al., 2020). In Iran, the most common mechanism of TBI is traffic trauma 41.4%, then war injuries 15.6%, and falls 8.8% (Heydari et al., 2019). A study in Latin America showed that traffic trauma is the most common cause of TBI in the population between 15 and 35 years of age (Dunne et al., 2020). Future research should be focused on the prevention of traffic trauma in the younger population and falls in the older population.

Considering the manner of injury, there is very rarely an isolated TBI. These are usually polytraumatized patients with injuries to the locomotor system, cervical spine, chest and abdomen. In the total sample, we had 37.1% of patients with TBI who also had polytrauma. In the study by Riemann et al. (2023), 4,225 patients were included, 164 (4%) patients had simultaneous spinal injuries, namely cervical 63%, thoracic 31%, mortality after 6 months was 11%, and only 36% of patients had a complete recovery. A study by Marchesini et al. (2024) of 1,545 patients had an associated C1–C2 cervical vertebral injury in 22 (1.4%) patients. There are still no clear views on the impact of extracranial injury on the clinical course and outcome of TBI. Niemeyer et al. (2022) concluded that the degree of mortality is predominantly related to the severity of TBI, regardless of extracranial injuries, while the results of another study show that mortality in isolated TBI is 17.8%, and in TBI with polytrauma is 21.8% (Watanabe et al., 2018).

Comorbidities in patients with traumatic brain injury can negatively affect the course of recovery and lead to long-term disability (Hanafy et al., 2021). Patients with more comorbidities were associated with approximately twice the likelihood of dying in the acute phase (Dell et al., 2021). Diabetes mellitus is a risk factor for longer hospitalization and stay in the Intensive Care Unit, and the increased level of glucose in serum in TBI patients is associated with an increased mortality rate (Tseng et al., 2023). In a 10-year study by Huang et al. (2022) that included 1,782 patients with TBI, it was concluded that mortality was significantly increased at a blood pressure < 90 mm Hg and above 190 mm Hg, while values between 130 and 149 mm Hg had a lower odds ratio of mortality. In a study by Dahdah et al. (2016) on 126 patients, the incidence of asthma was 6.4%. In our study, there is a higher number of comorbidities than in other studies, which can be explained by the older population of our sample. Data on the influence of comorbidities on the course and outcome of TBI have not yet been sufficiently examined, thus new studies that will shed light on their influence on the outcome of TBI are necessary.

In our study, at discharge from the Intensive Care Unit, there was no statistical difference between the groups, and at discharge from the hospital, 9.7% of patients in the experimental group had a tracheostomy, and 25.8% in the control group. The difference between the groups at discharge from hospital is statistically significant (*p* = 0.017), but significance has been lost after FDR correction (*p* = 0.170). In the acute phase of TBI, tracheostomy is often indicated, which reduces respiratory resistance, the need for sedation, shortens intubation time and intubation complications, and facilitates mechanical ventilation. On the other hand, the presence of a tracheostomy represents discomfort for the patient, makes verbal communication impossible, increases the risk of infection, makes the movements of the larynx difficult and increases the difficulty in swallowing, which is already impaired by TBI (Finotti et al., 2021).

In a one-year study in Germany, tracheostomy was performed in 23.1% of patients, and in a two-year study in 82.47% of patients with severe TBI (Intiso et al., 2017; Schwenkreis et al., 2021). As it can be seen from the data of various studies, the incidence of tracheostomy is very different, primarily depending on the severity of the traumatic injury and the duration of mechanical ventilation. The conclusion of the meta-analysis by Defranca et al. (2020) is that early tracheotomy in severe TBI increases the patient’s ability for early rehabilitation, thus improving the outcome . A retrospective study by Zivi et al. (2018) on 66 patients divided into two groups (group with early rehabilitation and with delayed rehabilitation in ICU), showed that early rehabilitation shortens the time to decanilman. In a prospective study by Zhou et al. (2022), it was concluded that intensive respiratory kinesitherapy enables decanilman in patients with prolonged tracheostomy.

At discharge from the ICU, all the patients from both experimental and control group had the presence of a urinary catheter and diapers, and at discharge from the hospital, the presence of a urinary catheter was more common in the control group in 77.4% compared to the experimental group in 61.3% of respondents. The difference was at the limit of statistical significance (*p* = 0.051), but significance has been lost after FDR correction (*p* = 0.204). A urinary catheter is placed for urinary dysfunction that develops after moderate and severe TBI, as well as for urinary tract monitoring. During treatment, spontaneous urination is usually established, but cases of hyperactive bladder and disorders of the lower urinary tract have also been described. A literature review by Wyndaele and Wyndaele (2023) all patients but one had an indwelling catheter on discharge from ICU. In the study by Bartolo et al. (2016) in Italy, 94.3% of patients had a urinary catheter at discharge from the ICU.

As for the presence of diapers, it was also more common in the control group 64.5% compared to the experimental group 46.8%. There is a statistically significant difference (*p* = 0.047), but significance has been lost after FDR correction (*p* = 0.204). In the study by Pelizzari et al. (2023) which included 521 patients with severe TBI at discharge, 53.3% of patients had fecal incontinence, there was a statistically significant correlation between incontinence and frontal lesions. Fecal incontinence should be seen as a consequence, not as a factor of an unfavorable outcome (Pelizzari et al., 2023).

Patients with moderate and severe TBI are at high risk of bed rest complications, such as decubitus ulcers, for this reason prevention measures are very important (Saint-Preux et al., 2021). In a study by Yoon and Cho (2022) on 237 patients, the incidence of decubitus ulcers was 13.9%, and in a study by Osis and Diccini (2020) on 240 patients, the incidence was 18.8%, i.e., 2.7, 23.2, and 42.6% in mild, moderate, and severe TBI. The conclusion of both studies is that the predisposing factors for the occurrence of decubitus ulcers are the severity of TBI, the use of noradrenaline, the use of mechanical ventilation, febrility and older age (Osis and Diccini, 2020; Yoon and Cho, 2022). Prevention measures include the positioning and application of an anti-decubitus mattress. A preventive as well as a multidisciplinary approach to positioning is of great importance in order to avoid complications and improve the quality of life after discharge. Positioning implies the position of the upper limbs in abduction at an angle of less than 90 degrees, the lower limbs must not be in hyperextension or in abduction of more than 30 degrees, ankle joints in a neutral position, trunk at an angle of 15 to 30 degrees. Scales for the early identification of patients at risk of developing decubitus ulcers are accepted worldwide (Ippolito et al., 2022). In our study, there were no decubitus ulcers in the experimental group at discharge from the ICU, while 4 (6.5%) patients from the control group had decubitus ulcers. The difference is statistically significant (*p* = 0.042), but significance has been lost after FDR correction (0.204). The incidence of decubitus ulcers in the ICU, according to the works published so far, is 13.9 to 18.8% (Osis and Diccini, 2020; Yoon and Cho, 2022). From our results, it can be concluded that extended early rehabilitation had a significant effect on reducing the incidence of decubitus ulcers in the ICU. At discharge from the hospital in the control group, decubitus ulcers were present in 8.1% of patients, and in the experimental group in 6.5%, there was no statistically significant difference (*p* = 0.729). Results of studies from Korea on 315 patients with severe trauma, decubitus ulceration occurred in 13.3% of patients, the average time of occurrence of decubitus ulceration was 9.74 days (Lim et al., 2023). In a systematic review Ribeiro et al. (2024) the incidence of decubitus ulcers varied between 6.5% and 20% among the included studies. According to the results of a study from Switzerland, the predilection places for the occurrence of decubitus ulcers are the heel 36% and the sacrum 29%, the presence of decubitus ulcers has a negative effect on functional recovery and increases the average length of hospitalization (Alito et al., 2023). According to the results of our research, both standard and extended early rehabilitation reduce the incidence of decubitus ulcers.

In our research, unadjusted regression models indicated a potentially beneficial effect of prolonged rehabilitation on certain clinical outcomes. The results of adjusted models emphasize the need for caution in interpreting differences between groups, given the variables selected in the study, as well as the sample size and construction. By applying scales that more accurately measure the outcomes of standard and extended rehabilitation treatment (Bartel index and Functional Independence Measure) on a larger sample, by applying adapted models, a more accurate detection of rehabilitation outcomes and differences between observed groups is expected. Minimal detectable change and minimal clinically important difference values provide thresholds for determining whether observed changes in outcome measures represent clinically meaningful improvements in TBI (Shen et al., 2025).

Although it has been in use for almost fifty years, GCS is irreplaceable in the clinical classification of brain injury severity and as a predictor of treatment outcome (Kodeeswaran et al., 2017; Ndoumbe et al., 2018; Swaminathan et al., 2023). Low admission GCS is an independent predictive factor for early in-hospital mortality and worse outcome in patients with TBI (Ndoumbe et al., 2018; Pastor et al., 2023). In our study, no statistically significant difference was found between the experimental and control groups in the GCS results at admission and discharge. However, a statistically significant difference was recorded in the analysis of the differences between admission and discharge in both groups (*p* < 0.001). Numerous authors emphasized age as one of the factors for a worse outcome measured by GCS (Kodeeswaran et al., 2017; Yamagami et al., 2019; Skaansar et al., 2020; Ogolo and Ibe, 2021; Neto et al., 2023). Studies that followed recovery after a longer period (3, 6, and 12 months) showed a better outcome measured by GCS (Sadaka et al., 2018; Nourelahi et al., 2022).

During the implementation of early rehabilitation, it is necessary to follow safety criteria that demonstrate cardiorespiratory function. Safety criteria for early rehabilitation include: heart rate of 60–130 per minute, systolic arterial pressure values of 90–180 mm Hg, mean arterial pressure of 60–100 mm Hg and blood oxygen saturation of > 88% (Sommers et al., 2015; Jang et al., 2019).

In our research, in the total sample, 91.1% of patients did not have an early rehabilitation interruption. The reasons for early rehabilitation interruption were: in 4.9% febrile, in 2.4% hemodynamic instability and in 1.6% worsening of the general condition (Keleman, 2024). In a meta-analysis by Nydahl et al. (2017) that included 48 publications with 7,546 patients and 23,351 procedures, there were adverse events 583 (2.6%). It can be concluded from the above that extended, individually dosed early rehabilitation is safe.

In our study, death was recorded in 8.1% of patients. In central Norway the overall mortality rate is 3.4/100,000 inhabitants, the percentage of deaths in patients with severe TBI was 49% in patients aged 60–69 years and 81% in patients aged 70–79 years (Pantelatos et al., 2023). In Australia, in the period from 2015 to 2020, the mortality rate was 14.9% in moderate and severe TBI (Reilly et al., 2023). According to studies published so far, mortality in moderate and severe TBI is from 6.9% in Belgium to 27.6% in Brazil, and in the Republic of Srpska during 2022 the mortality was 18.5% (Laiz et al., 2022; Niemeyer et al., 2022; Keleman et al., 2023). In a prospective multicenter study conducted in China from 2014 to 2017 data on the outcome and survival rate after TBI were analyzed. The study included 13,138 patients from 52 hospitals in 22 provinces in China. A total of 637 (5%) patients died, including 552 (20%) patients with severe traumatic TBI, and in another study from China with older patients and a fall as the leading mechanism of injury, mortality was 8.24% (Gao et al., 2020; Yang et al., 2022). In our research, there were a total of 10 (8.1%) deaths in both groups, five in both groups. No statistically significant difference was observed (*p* = 1.000). The study by Hayashi et al. (2025) showed that early rehabilitation did not affect mortality in TBI.

## 6 Limitation

This study has its limitations, which are primarily reflected in the small number of subjects, the short follow-up period, and the age of the study sample. It would be necessary to follow-up after 3, 6, and 9 months, because only after this period and extended inpatient rehabilitation is greater motor and cognitive recovery expected. Future research should address the creation of guidelines in the early rehabilitation of patients with TBI and answer questions about the exact duration of kinesitherapy treatment and the type of therapeutic interventions.

## 7 Conclusion

The optimal dosage, duration and frequency of kinesitherapy during the early rehabilitation of patients with TBI is a key problem today. Globally, there is no “gold standard” in the implementation of early rehabilitation interventions regarding the type of therapeutic intervention, its frequency, intensity and duration. It is recommended that early rehabilitation must be individually dosed, with a multidisciplinary approach. In our research, the patients of the control group had kinesitherapy twice for 30 min, and the patients of the experimental group twice for 45 min. According to the results of our research, extended early rehabilitation is safe, effective and contributes to improving treatment outcomes.

## Data Availability

The original contributions presented in this study are included in this article/supplementary material, further inquiries can be directed to the corresponding author.
